# Altruistic decisions are influenced by the allocation of monetary incentives in a pain-sharing game

**DOI:** 10.1371/journal.pone.0213104

**Published:** 2019-03-06

**Authors:** Ye-Seul Lee, Hyun-Seo Song, Hackjin Kim, Younbyoung Chae

**Affiliations:** 1 Acupuncture and Meridian Science Research Center, College of Korean Medicine, Kyung Hee University, Seoul, Republic of Korea; 2 Department of Anatomy and Acupoint, College of Korean Medicine, Gachon University, Seongnam, Republic of Korea; 3 Department of Psychology, Korea University, Seoul, Republic of Korea; Middlesex University, UNITED KINGDOM

## Abstract

**Background:**

Altruistic behavior is essential to the sustainability of society, but our current understanding of its underlying motivation is limited. In addition to the intrinsic motives to help others, based on empathy, extrinsic motives such as monetary incentives and social reputation influence prosociality. The purpose of this study was to examine the underlying motivations of prosocial behavior under constant or increasing extrinsic motivation settings.

**Methods:**

An experimental task, Altruistic Pain Sharing, was developed in which the participants were asked to share the other participants’ pain. In the session with monetary incentives, the incentives were given either constantly (CONSTANT condition) or proportionally (INCREASING condition), to the amount of shared pain. In addition, monetary incentives were not provided in the NO session. The participants experienced different amounts of mechanical pain at the beginning of the task and chose the number of pain stimulations to share, based on their experiences.

**Results:**

Compared to the NO session, the INCREASING session exhibited a rise in the mean of shared pain, but not the CONSTANT session. Furthermore, there was a distinct tendency to receive less pain than the other participant in the CONSTANT session, and a tendency to receive more pain than the other participant in the INCREASING session.

**Conclusion:**

Prosocial behavior was influenced by the presence, as well as the form, of the extrinsic monetary incentives. Our study shows that rewards incentivize individuals to demonstrate a higher level of prosocial behavior, implying that prosocial behavior is itself a mixture of intrinsic and extrinsic motivations, and that an effectively designed rewards system may function to enhance prosocial behavior.

## Introduction

A key component of the sustainability of society is prosocial or altruistic behavior[1]. The value and care that humans invest in each other’s welfare provides the basis for interpersonal relationships and cooperation, as well as the foundation for some professions such as those providing health care[2]. Current understanding of the underlying motivation for altruism shows that the ability to empathize may be essential when it comes to helping others at one’s own cost[3]. Previous studies have explored possible mechanisms and intrinsic motivations behind altruism, i.e., empathy and state of mind, as intrinsic mechanisms that enable altruistic decisions by allowing the individual to feel what the other person feels, and also the ability to represent what other people believe, even when those beliefs are mistaken or false[4, 5]. Taking in others’ distress intrinsically motivates individuals to share the distress, at the expense of one’s own comfort.

Interestingly, previous studies show that altruistic decisions often involve external pressures and incentives. Some studies have proposed that external incentives, such as social reputation and fairness, play a role in the presentation of prosociality[4, 6]. Altruistic behavior is not only socially desirable but also allows an individual the opportunity for self-presentation[7]. Social fairness has been agreed to be an inherent human trait of cooperation, and such altruistic preferences emerge in early life[8, 9]. In addition, not only altruistic decisions but also empathy itself may manifest themselves differently in altruistic behaviors depending on the socioeconomic status of the individuals concerned[10]. Furthermore, the provision of monetary rewards, as compared to giving pain, for the making of altruistic decisions[11] has been explored in an experimental setting looking at altruistic decisions[11]. Experimental approaches observed these prosocial and altruistic behaviors and the influence of external incentives through modified versions of solidarity games, such as the dictator game[4, 7, 12, 13], and tasks comparing altruistic behavior between pain and money[11]. In real-world settings, different forms of monetary rewards in health insurance, for instance, lead to changes in the number of medical services to patients [2, 14–18], opening up the discussion on moral hazards[19, 20] in relation to professionals such as health care providers.

These studies provide a deeper understanding of the motives behind altruistic behaviors. However, there seems to be a dichotomy between the inherent capability of humans to make altruistic decisions and the external incentives that promise rewards. We still do not fully understand the transition between two types of decision-making within one individual: the decisions understood as altruistic arising from empathy, and those understood as reward-seeking under extrinsic incentives or risk-avoiding under the external pressures. For instance, a recent study focusing on human behavior regarding energy conservation reported that nonprice information strategies motivated conservation behavior, while monetary savings information rather increased energy consumption[21]. This change of behavior implicates the transition from altruism to self-interest, due to framing effects in environmental decision-making in which the signaling of dollar savings linked energy consumption as a matter of an individual’s rewards[22]. In the case of medical doctors, studies show that the heterogeneity of the individual doctors, e.g., from different medical schools and medical specialties, predicts their level of altruism[23, 24]. Furthermore, this leads to moral hazard in health reimbursement systems, with repercussions appearing among doctors from different health care systems, whose decision may be based on self-interest[14, 18]. Recognizing this gap between the altruistic role of doctors and their reimbursement behavior, it can be argued that what has been considered a moral hazard may simply be pursuit for reward, manifested differently according to the provided external incentives.

The current study employed an experimental approach to study the decision-making of individuals in pain-sharing and monetary reward contexts. This study assessed whether (i) the presentation of external incentives and otherwise unchanged conditions influences the pain-sharing decision; (ii) the allocation of external incentives relative to pain sharing influences the pain-sharing decision; and (iii) whether the influence of external incentives varies according to decisions based on altruism. We hypothesized that pain-sharing behavior is influenced differently not only by the presentation of external incentives but also by the allocation of incentives to share pain. We also hypothesized that the external incentives have a greater influence on individuals who choose to share less pain altruistically.

## Methods

### Participants

Forty participants (mean age 23.8 ± 4.3 years, 22 males) took part in the study. The title of the experiment that was used for recruitment was, “The inter-relationship between economic valuation and somatic sensation.” They had no known history of neurological, psychiatric or chronic pain disorders. After providing the participants with information about the nature of the experiment, the participants provided full written consent. All participants received a detailed explanation of the study and written, informed consent was obtained. The study was conducted in accordance with the Declaration of Helsinki and was approved by the Institutional Review Board at Kyung Hee University (KHSIRB-17-046).

### Experimental design

**Fig 1:**
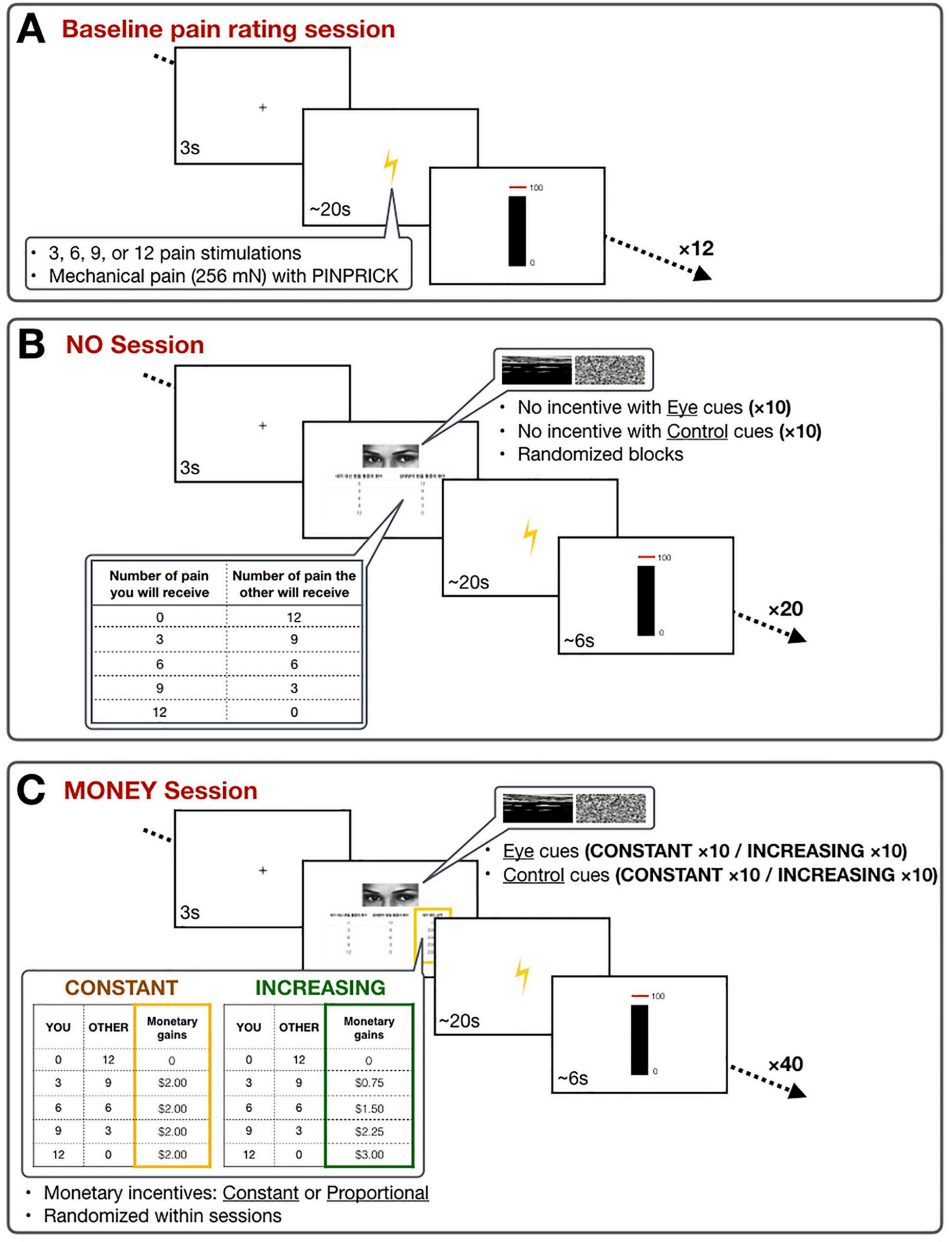
Experimental protocol. **A.** Baseline session. The participants received stimulations (3, 6, 9, or 12) of mechanical pain and evaluated the pain intensity in a visual analogue scale (VAS: 0 –not painful at all; 100 –as painful as one can imagine). **B.** NO session. The participants were asked to choose how many of the pain stimulations, to be received by the other participant, that they would share. Subtle eye cues or control cues were presented above the choice table. After choosing the number of stimulations, the participants received the chosen number of pain stimulations. **C.** MONEY session. Similar to the NO session, the participants were asked to choose how many of the pain stimulations, to be received by the other participant, that they would share. In addition, the choice table included two types of monetary incentives: constant or increasing. Subtle eye cues or control cues were presented above the choice table. After choosing the number of stimulations, the participants received the chosen number of pain stimulations.

To observe the participants’ prosocial or other-regarding behavior, we developed an experimental approach called the Altruistic Pain-Sharing Task. In this task the participants were asked to share the other participant’s pain with and without monetary incentives (NO session and MONEY session), and in the MONEY session, constant or proportionally increasing incentives to the amount of shared pain (CONSTANT condition and INCREASING condition). In addition to the explicit incentives within the study, implicit or subtle cues were included in the experiment as either eye or scrambled images on the screen without providing any information regarding the images prior to the experiment[12, 25]. The distribution of monetary incentives was presented in pseudorandom order within blocks, and eye/control cues were divided into blocks in random order in the NO and MONEY sessions. The experiment was a within-subject study with 3 (incentive types: NO, CONSTANT, INCREASING) × 2 (social cues: eye, scramble) design (Fig 1). The participants’ responses were recorded using “Psychtoolbox” in MATLAB.

### Experimental procedure

An experimental approach named the Altruistic Pain Sharing Task was developed for this study. In this experiment, the participants were given instruction in pairs, one of whom was a confederate, a female whom the participant had not met before. The pain sharing task in this experiment was introduced to the participants under the title of “A study on the exchangeability between pain sensation and monetary values.” The task required the participant to choose the number of pain stimulations that they would take from the pain of the other participant (confederate) in exchange for monetary rewards. Out of the twelve stimulations that the other participant would receive, the participant had to choose among 0, 3, 6, 9, and 12 stimulations, to share the pain. Participants were also told that there were two different scenarios in which they had the decision: 1) no monetary incentives, and 2) monetary incentives for the shared pain. The participants were told that the final sum of money from the decisions that they have made will be reflected to the participation payment at the end of the experiment. They were instructed to evaluate the intensity of pain at the end of each trial. After the instruction, the confederate left the instruction room first, followed by the participant, who was guided to another room where the experimenter was waiting.

### Altruistic pain-sharing task

#### Baseline pain-rating session

In the baseline session, the participants were given different sets of numbers of mechanical pain stimulations (Pinprick stimulators: MRC Systems GmbH, Heidelberg, Germany) on the lateral proximal side of their left forearm. The baseline session was always done at the beginning of the experiment to allow the participants to estimate the degree of pain the other participant would receive, thereby providing them the information they would need before deciding how much pain they will share. The intensity of pain was kept to 256mN throughout the experiment, and the number of painful stimulations in one trial was varied, between 0, 3, 6, 9, and 12. Each number of painful stimulations was repeated three times. The participants were asked to rate the pain intensity using visual analogue scale (VAS: 0 –not painful at all; 100 –as painful as one can imagine) (Fig 1A).

#### Monetary incentives conditions

The NO session and the MONEY session were divided into blocks (6 blocks in total; 2 blocks of NO session, 2 blocks × 2 conditions of MONEY session; 20 trials in each session). The order was pseudo-randomized among the participants so that exactly half the participants went through the NO session prior to the MONEY session, and the other half went through the MONEY session prior to the NO session. Within the MONEY session, CONSTANT and INCREASING conditions were randomized. In the NO session (Fig 1B), the participants faced the choices of 0, 3, 6, 9, and 12 painful stimulations that they had to receive for the other participant. Once they had decided the number of painful stimulations, the participant received mechanical pain, and the intensity was identical to that in the baseline stimulation. In the case where the participant made choices of 0 or 3 painful stimulations, the experimenter took time after the painful stimulation to give the impression that the other participant was receiving pain. After the stimulation, the participants evaluated the intensity of the pain.

In the MONEY session (Fig 1C), there were two different conditions. The CONSTANT condition provided constant monetary rewards for the shared pain ($2.00 regardless of the amount of shared pain), and the INCREASING condition provided proportionally increasing monetary rewards (0 shares—$0; 3 shares—$0.75; 6 shares—$1.50; 9 shares—$2.25; 12 shares—$3.00). The amount of monetary gain was tagged to each choice according to different conditions. The order of the two conditions was pseudo-randomized within the blocks so that no more than two identical cues appeared in a row. Upon seeing the choice table, the participant decided the number of painful stimulations to share out of 5 choices (0, 3, 6, 9, and 12 pain shares), with the same procedure in the NO session. After the painful stimulation, the participants evaluated the pain intensity.

#### Social reputation cues

In addition to the different conditions of the monetary incentives, the screen either showed eye or scrambled images above the choice tables. Since the eye condition was designed to represent implicit social monitoring during the task, no additional verbal or printed instruction regarding eyes, social monitoring, or social reputation was provided to the participants. The eye and control cues were presented in both the NO and MONEY sessions, and the orders of the cues were randomized as blocks nested within NO and MONEY sessions. The eye image from the “reading the mind in the eyes” test that is most neutral in terms of emotion was selected[26, 27] for the experiment, so as to use eyes from the picture of a human. For the control condition, the same image was scrambled using the “JigSaw” function in MATLAB.

#### Post-experiment surveys

After the experiment, the participants were required to fill out an empathy quotient (EQ) questionnaire and a Social Desirability Scale (SDS) questionnaire. The EQ questionnaire was developed by Baron-Cohen et al.[28] to measure empathic characteristics of adults of normal intelligence. This questionnaire consisted of 40 empathy questions and 20 filler/control questions, giving a maximum achievable score of 80. Kim et al[29] translated the questionnaire into Korean and validated the translated questionnaire. We used the validated Korean version of the EQ scale. The SDS questionnaire was first developed by Crowne and Marlowe [30] to measure the need of individuals to obtain social approval. The original SDS questionnaire consists of 33 items on a personal reaction inventory. Kim et al.[31] translated the questionnaire into Korean and validated the shortened questionnaire. We used the validated Korean version of the SDS scale in this study.

### Statistical analysis

The main outcome measure was the amount of shared pain in the different conditions of the Altruistic Pain-Sharing task. Statistical analysis included 3 (condition: NO, CONSTANT, INCREASING) × 2 (condition: eye, scramble) analyses of variance (ANOVA). In addition, to examine the potential gender effect in the altruistic choice, an additional 3 (condition: NO, CONSTANT, INCREASING) × 2 (condition: male, female) analyses of variance (ANOVA) was conducted. Furthermore, to observe the change of behavior by the allocation of monetary incentives, the pain shares were categorized as “less than”, “same as”, and “more than” the other participant, to analyze the participant’s decision on the basis of altruism. The pain-sharing decisions were analyzed using linear regression in the three conditions independently. Next, to analyze whether pain-sharing decisions changed across conditions, the β values or the regression coefficients were compared between conditions for statistical differences by computing regression models with an interaction term between condition and altruism, and by using analysis of variance (ANOVA) between regression models[32, 33].

To determine the effect of incentives on the change of altruistic behaviors in the Altruistic Pain-Sharing task, correlation analyses were performed between the individual mean of shared pain in the NO session and the individual mean of shared pain in the MONEY session. Furthermore, another correlation analysis was performed between the individual mean of shared pain in the NO session and the individual differences between the INCREASING and CONSTANT conditions. In addition, a correlation analysis between the participants’ empathy and SDS, and their behavior in the task was performed.

Lastly, a regression model was created to understand the factors that influenced the altruistic choice of the participants. The factors tested in this experiment that may have influenced altruistic decision were monetary conditions, eye cues, gender, the individual’s average pain ratings and pain sensitivity during baseline pain stimulation, and post-experiment questionnaire responses. The average pain ratings of the individuals were calculated across different pain stimulations, and pain sensitivity was evaluated as the regression coefficient of the individual participant’s ratings to different painful stimulations in the baseline session. The shared pain was categorized as not altruistic when they received fewer painful stimulations than the other, fair when they received the same number, and altruistic when they received more than the other. The distribution of the responses was a Poisson distribution.

Among the variables, monetary conditions and eye cues were fixed; the individual’s average pain ratings and pain sensitivity and the questionnaires, on the other hand, were random effects. Considering that there were both fixed and random effects, and that the responses formed a Poisson distribution, a generalized linear mixed model (GLMM) was chosen for the final modeling. Among the possible model including the variables tested in the experiment, the final model chosen for the highest predictive accuracy and after comparing the goodness-of-fit through Akaike information criterion (AIC), Bayesian information criterion (BIC), and log likelihood, included the fixed effects except for gender and random effects except for the questionnaires including SDS and EQ. The model was estimated and fitted by maximum likelihood or Laplace approximation. The final model is reported in Table 1 including fixed effects and their 95% CIs. All statistical analyses were conducted using R software (version 3.2.3 “Wooden Christmas-Tree,” http://r-project.org/), with *p* < 0.05 representing statistical significance.

**Table 1:** Fixed effects of monetary conditions and eye cues in the GLMM model.

Explanatory variables	Coefficient	95%CI	p-value
Money (fixed effect)	0.279	[0.095, 0.465]	< 0.001
Eye cues (fixed effect)	0.02	[-0.164, 0.205]	0.820
Individual pain sensitivity (random effect)	0.02	[-0.067, 0.027]	0.383
Individual pain intensity ratings (random effect)	-0.002	[-0.006, 0.002]	0.302

## Results

### Baseline pain ratings

The results from the experiment included data from 40 participants (18 females, 22 males). The baseline pain level that the participants initially received was analyzed. The average of the VAS ratings increased as the number of stimulations increased (3 stimulations of mechanical pain (Mean ± SEM): 32.2 ± 3.7, 6 stimulations: 42.7 ± 3.6, 9 stimulations: 47.6 ± 3.8, 12 stimulations: 50.9 ± 4.1, 1-way ANOVA main effect: F = 13.22, p < 0.001).

### Shared pain in Altruistic pain-sharing task

**Fig 2:**
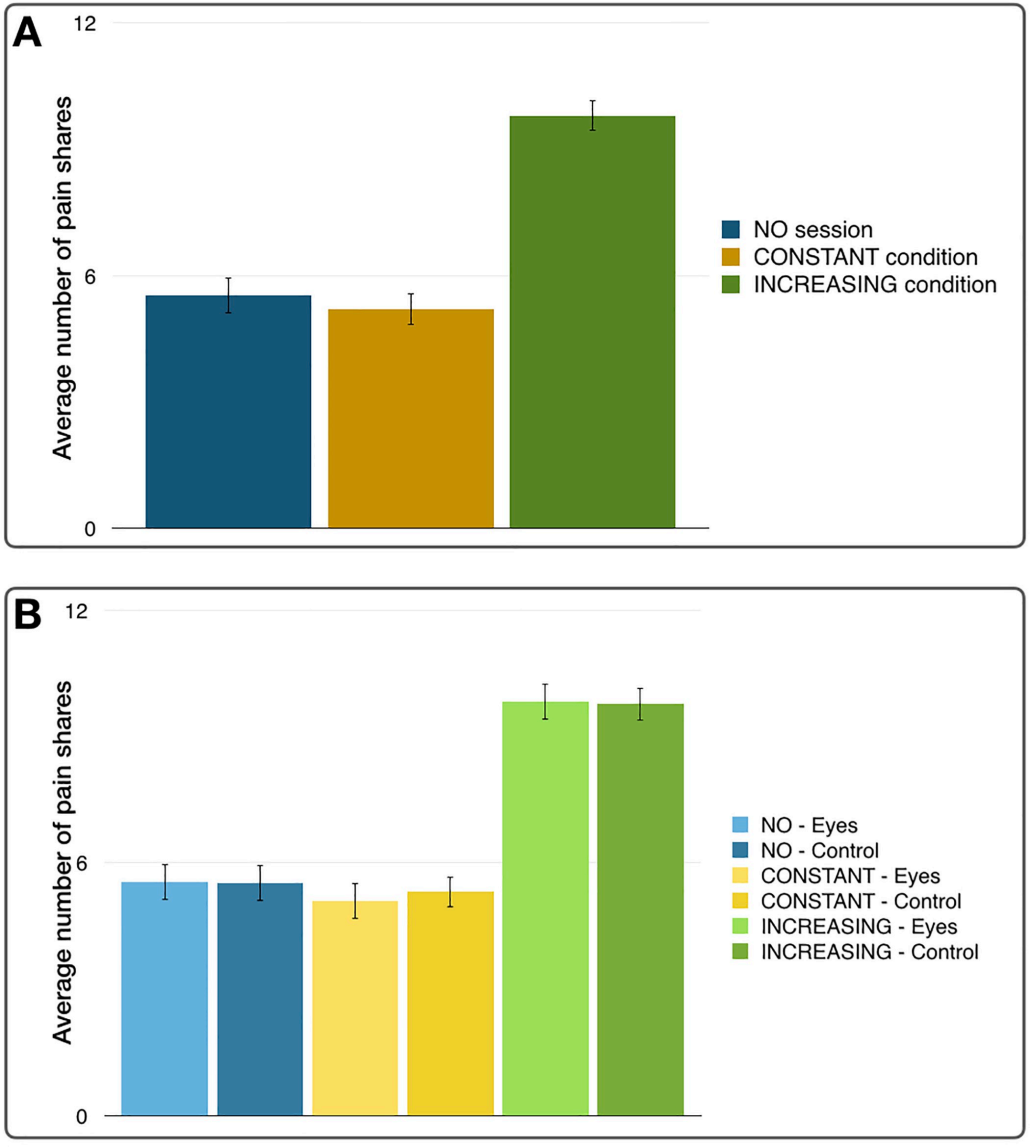
**A.** Average number of shared pain stimulations by session. It increased during the INCREASING session, while there was no significant difference between the NO session and the CONSTANT session. **B.** Average number of shared pain stimulations by session and by eye or control cues. There was no significant difference between eye or control cues throughout all sessions.

The shared pain levels in Altruistic Pain-Sharing task were 5.5 ± 0.4 in the NO session, 5.2 ± 0.4 in the CONSTANT condition, and 9.8 ± 0.4 in the INCREASING condition. The amount of shared pain showed significant differences between NO, CONSTANT, and INCREASING conditions (Fig 2A). ANOVA showed a significant effect of the monetary conditions by allocation, but no significant difference in the amount of shared pain between the Eye condition and control condition (Fig 2B; 2-way ANOVA, main effect: F(2, 234) = 0.064, p = 0.938, between the form of incentives: F(2, 234) = 79.193, p < 0.0001; between the Eye and control condition: F(1, 234) = 0.016, p = 0.898). Furthermore, the effect of the monetary conditions and the gender effect was examined in the amount of shared pain, which showed that while there was a significant effect of the monetary conditions by allocation, there was no gender effect observed in the amount of shared pain (3-way ANOVA, main effect of gender: F(1, 234) = 0.289, p = 0.591, between the form of incentives: F(2, 234) = 78.960, p < 0.0001; between the Eye and control condition: F(1, 234) = 0.016, p = 0.899, interaction between incentives and gender: F(2, 234) = 2.370, p = 0.096, interaction between eye/control conditions and gender: F(1, 234) = 0.138, p = 0.710, interaction between incentives and eye/control conditions: F(2, 234) = 0.064, p = 0.938, interaction among incentives, eye/control conditions, and gender: F(2, 234) = 0.070, p = 0.932).

### Altruistic choices between monetary conditions

**Fig 3:**
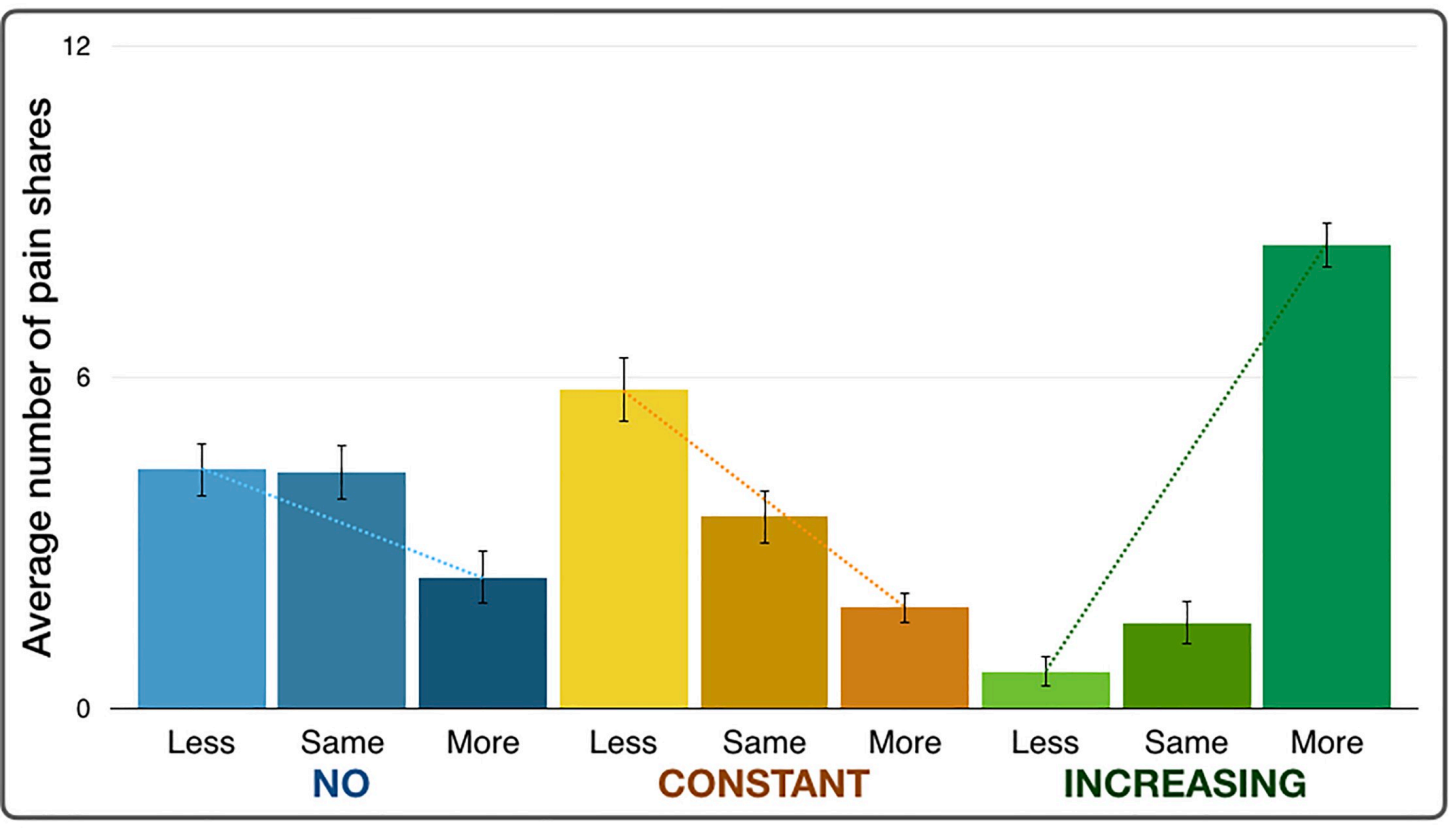
Average number of shared pain stimulations categorized by altruistic choices shows that during the CONSTANT session, there was a trend for sharing less or the same amount of pain. During the INCREASING session, there was a trend for sharing more pain.

We next categorized the choice of pain shares on the basis of altruism and on the overall average of shared pain in NO session which showed that participants shared about half of the other’s pain, specifically, as: “less than the other participant” (0 or 3 shared pain stimulations), “same” (6 shared pain stimulations), and “more than the other participant” (9 or 12 shared pain stimulations) as shown in Fig 3. The results showed that in the NO condition, participants decided to share the same or less than the other participant (β = -0.986, p = 0.006). In the CONSTANT condition, the tendency in the decisions for pain shares leaned toward less pain than the other (β = -1.967, p < 0.0001). Finally, in the INCREASING condition, participants decided to share more pain (β = 3.903, p < 0.0001). Comparison between regression coefficients across conditions using ANOVA between regression models showed that the regression coefficients or slopes were statistically different among the NO, CONSTANT, and INCREASING conditions (F = 42.819, p<0.001).

### Correlation analysis of pain shares between monetary conditions

**Fig 4:**
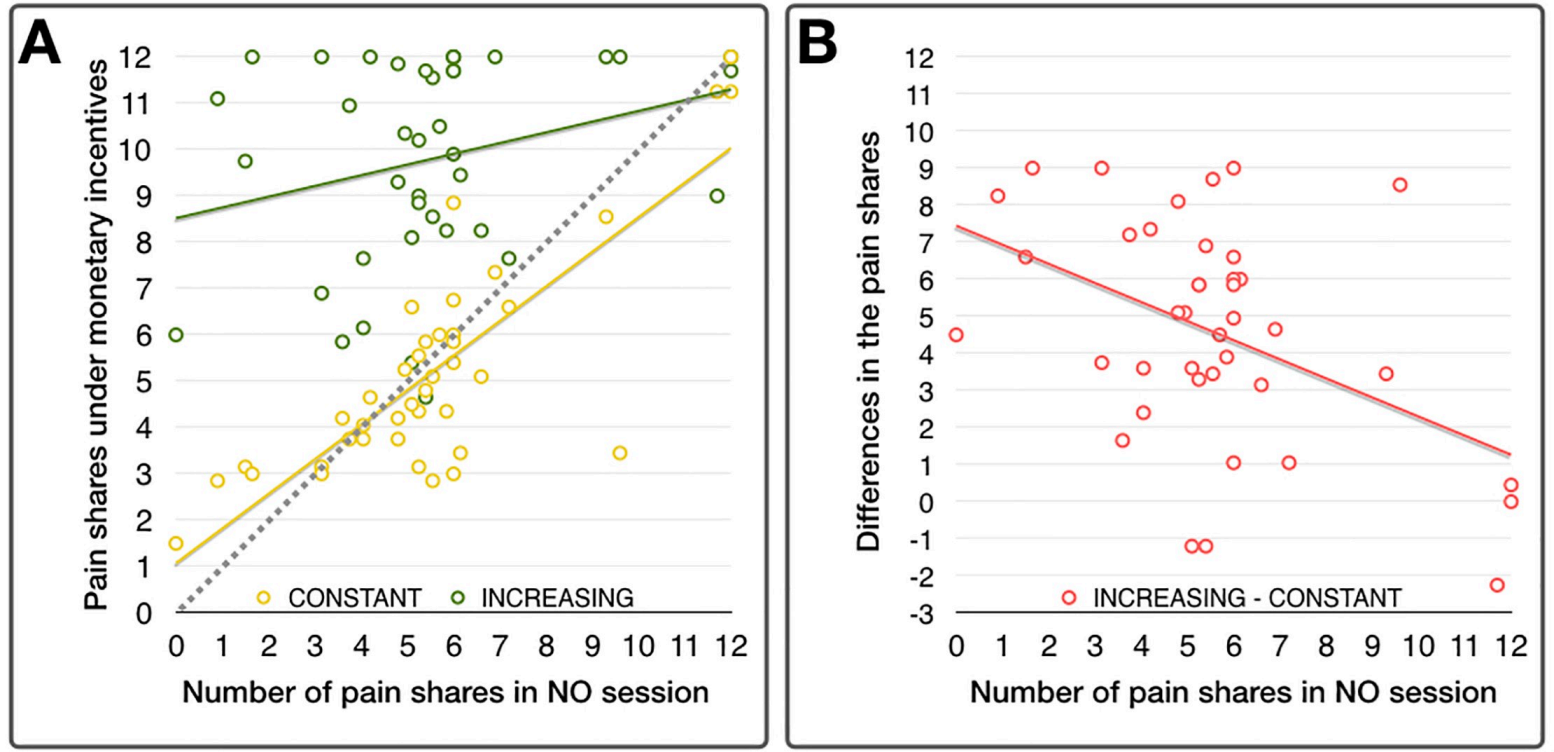
**A.** Scatterplot of the individuals’ average amount of shared pain during the CONSTANT session and the amount of shared pain during the NO session were significantly and positively correlated, while shared pain during the INCREASING session and during the NO session were correlated without significance. **B.** Scatterplot of the individuals between the average number of pain shares in the NO session and the differences between the CONSTANT and INCREASING conditions were significantly negatively correlated.

The levels of individual shared pain in the NO session and those in the CONSTANT condition were significantly correlated (r = 0.813, p < 0.0001), and the individual mean of shared pain in a NO session and those in an INCREASING condition showed positive correlation without significance (r = 0.274, p = 0.087) as shown in Fig 4A. The individual differences between the mean of shared pain in the INCREASING and CONSTANT conditions were also significantly negatively correlated (r = -0.449, p = 0.004, Fig 4B), implying that monetary incentives induced higher responses in the participants who initially made less altruistic decisions.

On the other hand, the participants’ choices in the experiment were not correlated with their EQ scores (EQ–No incentives session: r = -0.205, p = 0.205; EQ–CONSTANT condition: r = -0.015, p = 0.926; EQ–INCREASING condition: r = 0.019, p = 0.907). Similarly, the participants’ choices in the experiment were not correlated with their SDS scores (SDS–NO session: r = 0.171, p = 0.291; SDS–CONSTANT condition: r = 0.121, p = 0.456; SDS–INCREASING condition: r = -0.016, p = 0.922).

### Generalized linear mixed model (GLMM)

The altruistic responses were fitted with GLMM to construct a model that predicts altruistic decisions with high accuracy. After testing for overdispersion through residual distribution and constructing candidate models based on our experiment, the final model was selected for the lowest AIC and BIC. In the final model (Akaike information criterion (AIC) = 708.0, Bayesian information criterion (BIC) = 739.4, log likelihood = -345.0), the gender and post-experiment questionnaire responses were excluded (Table 1). The analysis of the altruistic responses showed that only the monetary incentives were significant in the overall model (*β* = 0.28, p < 0.001). The fixed effects of eye cues (*β* = 0.02, p = 0.820) did not influence the responses. Random effects such as the individual pain sensitivity and individual pain intensity ratings did not influence the responses (Table 1).

## Discussion

This study explored the effect of monetary incentives on the prosocial behavior of sharing another person’s pain. In the Altruistic Pain-Sharing task, the participants shared about half of another person’s pain without monetary incentives. When constant monetary incentives were provided, the average amount of shared pain did not change significantly; however, when proportionally increasing incentives were provided, the average amount of shared pain increased. The effect of the allocation of monetary rewards under the NO, CONSTANT, and INCREASING conditions had an effect on the tendency to share pain when the participants’ choices were categorized by altruism; participants in the CONSTANT session decided to share less pain more often than in the NO session. Conversely, the participants in the INCREASING session decided to share more pain than in both the NO and CONSTANT sessions. The correlation analysis of individual participants showed that the individuals’ decisions to share pain in the NO session were positively correlated with the amount of shared pain in the CONSTANT sessions, as well as in the INCREASING sessions. However, the differences in the levels of shared pain in the INCREASING and CONSTANT sessions were negatively correlated with the number of shared pain choices in a NO session.

The decision to share about half the painful stimulations with an individual whom the participants had met only once, without any monetary reward, represents altruistic behavior involving neither consequential rewards nor interpersonal closeness, as previously illustrated in a number of studies[34–36]. Note that the experimental game in our study followed a pseudo-randomized order, which revealed to the participants that the monetary incentives were absent in some sessions and present in others. In addition, it was confirmed that the participants in our study had never met the confederate prior to our study. Nonetheless, the individuals shared more than five out of twelve painful stimulations received by the other person they had met once, implying that this behavior was largely other-oriented[11]. Previous studies have proposed that the underlying mechanisms of altruistic behavior are social fairness and empathy[1, 3, 37, 38]. Empathy and theory of mind help to explain altruism on the basis that an individual is able to feel what another person is feeling in a given situation, as well as being able to represent what other people feel or believe, even though such representations may be mistaken[4, 5]. Social fairness has been revealed in previous studies to play an important role in donation, pain sharing, and altruistic punishment through the inherent human trait of collaboration and egalitarian motivation, which emerges in early life[8, 9]. In addition to social fairness, recent studies suggested that moral preference may influence altruistic behavior[39–41]. Further evidence suggests that empathy may manifest itself in altruistic behaviors differently, depending on the social environment[10].

Interestingly, the participants’ decision to share pain under monetary incentives was influenced by the allocation of monetary rewards to pain shares. When monetary rewards depended on the number of pain shares, participants’ pain shares increased. The willingness of the individuals to risk a higher intensity of pain to obtain a larger amount of money seems to be the result of reward-seeking behavior[38]. On the other hand, in sessions where monetary rewards were presented regardless of the number of pain shares, decisions to make altruistic choices showed a significant shift toward less pain shares than in sessions where no explicit rewards were presented but were otherwise identical. This shift may be due to the estimation of prospective rewards, rather than altruism, affecting the decision, and may indicate a transition of behavior from other-regarding or altruistic to reward-seeking, triggered by monetary rewards. In addition, the GLMM model suggested that while the presentation of monetary incentives had an effect in the participants’ decision to share pain, neither the individuals’ pain intensity ratings nor their sensitivity to pain seemed to have a significant effect in the decision. Taken together, it seems that while monetary rewards incentivize individuals to take greater degrees of pain, their influence on altruistic decisions may depend on the distribution of rewards, rather than merely the presence of the reward itself.

Previous studies show that the expected outcome of economic activity and its value influence the decisions of individuals in both behavioral and neural responses[42], and that contextual values of the reward significantly influence the risk preference[43]. In our study, the decision to receive greater amounts of pain in return for increasing monetary rewards implies that while the participants were aware of the risk of a painful experience, they were willing to risk the unpleasantness of the pain for a reward. On the other hand, the decision to receive less pain for the return of constant rewards showed that the participants chose to avoid risking more pain for the same amount of reward. Although the risk of gains and losses of monetary returns are different in context from the risk of receiving pain, the pursuit of reward is reported to be closely related to individuals’ decisions on risk preferences. This incentive-dependent behavior has been reported to elicit distraction in terms of its performance[44], and to alter the estimation of effort on receipt of reward[45].

Understanding altruism from both the intrinsic motivation of other-regarding behavior and the external incentives of monetary rewards, it is suggested that prosocial behavior or altruistic choices can be moderated through the effective presentation of rewards. Proportionally increasing incentives generally maximized the number of shared painful stimulations, and the responsiveness to monetary rewards was stronger in the individuals who were initially averse to take higher risks for constant reward. Previous studies also show that prosocial behavior is a mixture of responses to both external and intrinsic pressure and reward[46]. Extrinsic reward can influence or undermine a person's intrinsic motivation to engage in a task[47]. Furthermore, the role of extrinsic reward and intrinsic motivation in prosocial behavior can be applied to social sciences and public health, e.g., policies and financial settings influencing the behavior of health-care providers. Intrinsic motives can become confused with the presentation of extrinsic incentives, and differently designed health payment schemes lead to different amounts of health service to patients [2, 14–18]. What has been largely considered a moral hazard[19, 20] may be a response as an individual to extrinsic pressure and intrinsic motivations, a behavior in pursuit of reward, and the consequent decision to take more or less risks. It may be crucial for policymakers as well as principals, therefore, to design an effective setting for an agent to produce the most efficacious performance.

It is noteworthy that the design of the study included the subtle presentation of eyes to the participants through the screen; this changed neither the number of pain shares nor the categorized altruistic choices. Previous studies have effectively illustrated the effect of observability by the presence of others in the altruistic choices made by healthy participants[4]. The effect of the presence of others may also be related back to the influence of social reputation on altruistic behaviors, where the individuals are more prone to self-presentation effects. Similarly, eye cues have been used in several previous studies to explore the impact of observability on prosocial behavior, with mixed results[12, 25, 48, 49]. A possible explanation for the lack of effect from the eye cues in our study may be that the repeated presentation of eyes and control images, due to the within-subject design, may have dulled the effect of seeing the eyes on the screen. While the eye cues were presented on the screen in random order, there was no instruction prior to the experiment mentioning eye gazes to the participants. It also points to the difficulty of implementing social monitoring, which, in the previous study, relied on an excuse of a system breakdown during the experiment[4].

Lastly, previous studies suggest that there is a gender difference in the manifestation of altruistic behavior. Females have been reported to be more altruistic in Dictator games[50, 51], blood donations[52], and in their careers as physicians[53]. Furthermore, a recent study showed that women are well aware of the gender stereotype-based expectations[54], and this bias itself may motivate women towards more altruistic behaviors[55]. On the other hand, this study did not show a significant gender effect in sharing pain. This finding may be due to the nature of the experiment, where the participants were asked to share pain instead of donating money; and it may also be due to the gender of the confederate, who was a female. Further studies are required to understand the gender effect in pain sharing behaviors.

The limitations of this study include the difficulty in implementing social cues such as eye images during the experiment to increase observability. The effect of subtle eye cues would need to be explored in depth, and perhaps a further investigation on the effect of observability through cues other than a real observer or the eye cues, such as a camera, may add to the study of prosocial behavior. Another limitation is the order of the overall experiment, during which the questionnaires were always asked after completing the tasks, which may influence the responses to the questionnaire items. This order was inevitable as the tasks were first introduced to the participants as “A study on the exchangeability between pain sensation and monetary values,” thereby removing any suggestions regarding altruistic choices in the introduction and to rule out possible effects of social desirability. This order of the experiment allowed the participant to make decisions without being aware of the altruism in his or her own decisions. Lastly, this study explores the role of rewards in altruistic choices, and further studies regarding the role of risks in altruistic choices would provide deeper insights regarding altruism.

In conclusion, this study explores altruistic behavior and the impact of extrinsic rewards through a task developed for this experiment. The behavior of sharing pain was observed with and without monetary rewards, but the performance of altruistic behavior changed significantly according to the presence, as well as the distribution, of the rewards. Our study implies that rewards incentivize individuals to manifest a higher level of prosocial behavior, and that prosocial behavior itself is a mixture of both intrinsic motivations and extrinsic incentives. An effectively designed reward system may play the role of enhancing prosocial behavior as well as the performance of professionals such as health care providers in a specific medical setting.

## Supporting information

S1 Table Number of shared pains of each participant by monetary incentives conditions and social reputation cues. (CSV)
